# Supplementary material for: Spinal fMRI demonstrates segmental organisation of functionally connected networks in the cervical spinal cord: A test–retest reliability study
Source: Hum Brain Mapp. 2024 Jan 31;45(2):e26600. doi: 10.1002/hbm.26600 (PMC10831202; doi:10.1002/hbm.26600)
Supplement: Supplementary file 1 — Data S1: Supporting information. [file HBM-45-e26600-s001.docx]

***Supplementary Materials for***

**Spinal fMRI demonstrates segmental organisation of functionally connected networks in the cervical spinal cord: A test-retest reliability study**

**Table of contents:**

[1 Comparison high-tSNR and low-tSNR data 2](#_Toc154052017)

[2 Group average EPI image in PAM50 space 2](#_Toc154052018)

[3 Voxelwise connectivity analysis for data acquired during MRI session 2 3](#_Toc154052019)

[3.1 Methods 3](#_Toc154052020)

[3.2 Results 3](#_Toc154052021)

[4 Seed-to-seed connectivity analysis for data acquired during MRI session 2 6](#_Toc154052022)

[4.1 Methods 6](#_Toc154052023)

[4.2 Results 6](#_Toc154052024)

[5 Seed-to-seed connectivity analysis on unsmoothed data 8](#_Toc154052025)

[5.1 Methods 8](#_Toc154052026)

[5.2 Results 8](#_Toc154052027)

[6 Association between connectivity and ICC 10](#_Toc154052028)

[6.1 Methods 10](#_Toc154052029)

[6.2 Results 10](#_Toc154052030)

[References 11](#_Toc154052031)

# Comparison high-tSNR and low-tSNR data


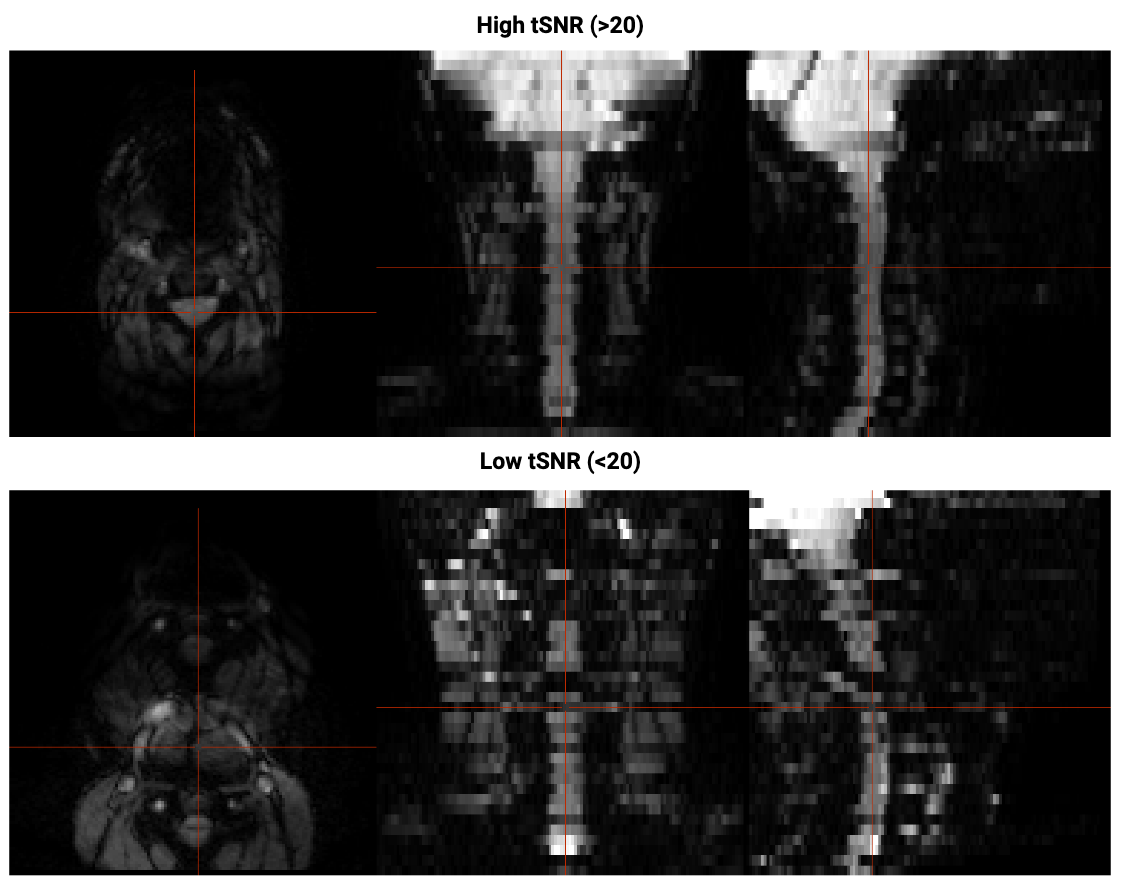


Supplementary Figure 1. Visual comparison of representative data from a subject characterised by high tSNR (>20; upper pane) and low tSNR (<20; lower pane).

tSNR = Temporal Signal-to-Noise Ratio.

# Group average EPI image in PAM50 space


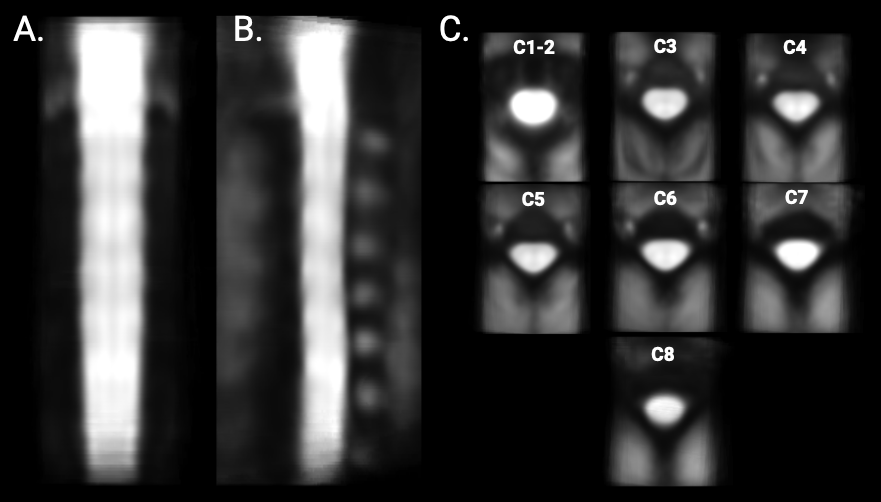


Supplementary Figure 2. A group mean EPI image in Polytechnique Aix-Marseille University and Montreal Neurological Institute 50 (PAM50) template space showing the average signal intensity in mid-coronal (A), mid-sagittal (B), and axial planes (C). A NIfTI file used to generate this figure is available for download from NeuroVault (<https://identifiers.org/neurovault.collection:13616>).

# Voxelwise connectivity analysis for data acquired during MRI session 2

## Methods

Seed-based voxelwise connectivity analysis of data acquired during MRI session 2 followed the same steps as that of MRI session 1 (described in the main text). Mean timecourses extracted from regions of interest (L/R DH/VH of each segmental level from C5 to C8) were used to provide voxelwise estimates of functional connectivity within the entire cervical cord. Subject-level modelling was performed in FEAT by including all four seeds’ timecourses (L DH, R DH, L VH, R VH) from a given segmental level (C5, C6, C7, C8) in one model. COPE images from this stage were registered to PAM50 space using warp parameters generated during preprocessing (see main text).

Spatial extent of resting-state networks at group level was assessed using *randomise* (Winkler et al., 2014) with threshold-free cluster enhancement (5000 permutations, *p*< 0.003 (*p*= 0.05, Bonferroni corrected for 16 individual seed regions)).

## Results

Resting-state networks observed on MRI session 2 were highly similar to those observed on MRI session 1 (see Supplementary Figure 2 for overlap between clusters). Networks were largely confined to each segmental level, with sparser between-segment connections (Supplementary Figure 3-6). Similar to the results of session 1 data analysis, we observed a dorsal bias in functional connectivity of dorsal seeds and a ventral bias in functional connectivity of ventral seeds.


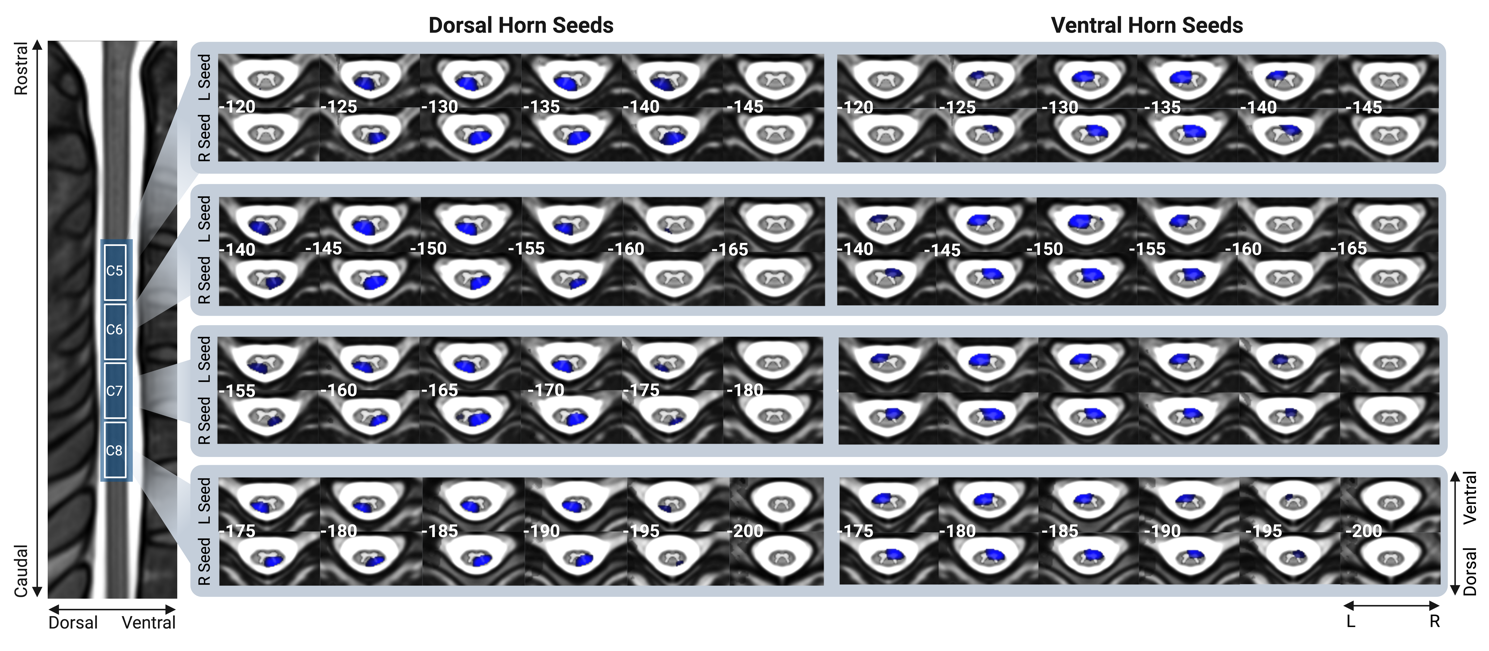


Supplementary Figure 3. Resting-state networks obtained from voxelwise connectivity analysis for each of the four quadrants (ventral/dorsal and left/right) of segmental levels C5-C8. Axial slices are marked with the *z* MNI coordinate. Each resting-state map was thresholded at *p*< 0.003 (*p*= 0.05, Bonferroni corrected for 16 individual seed regions).


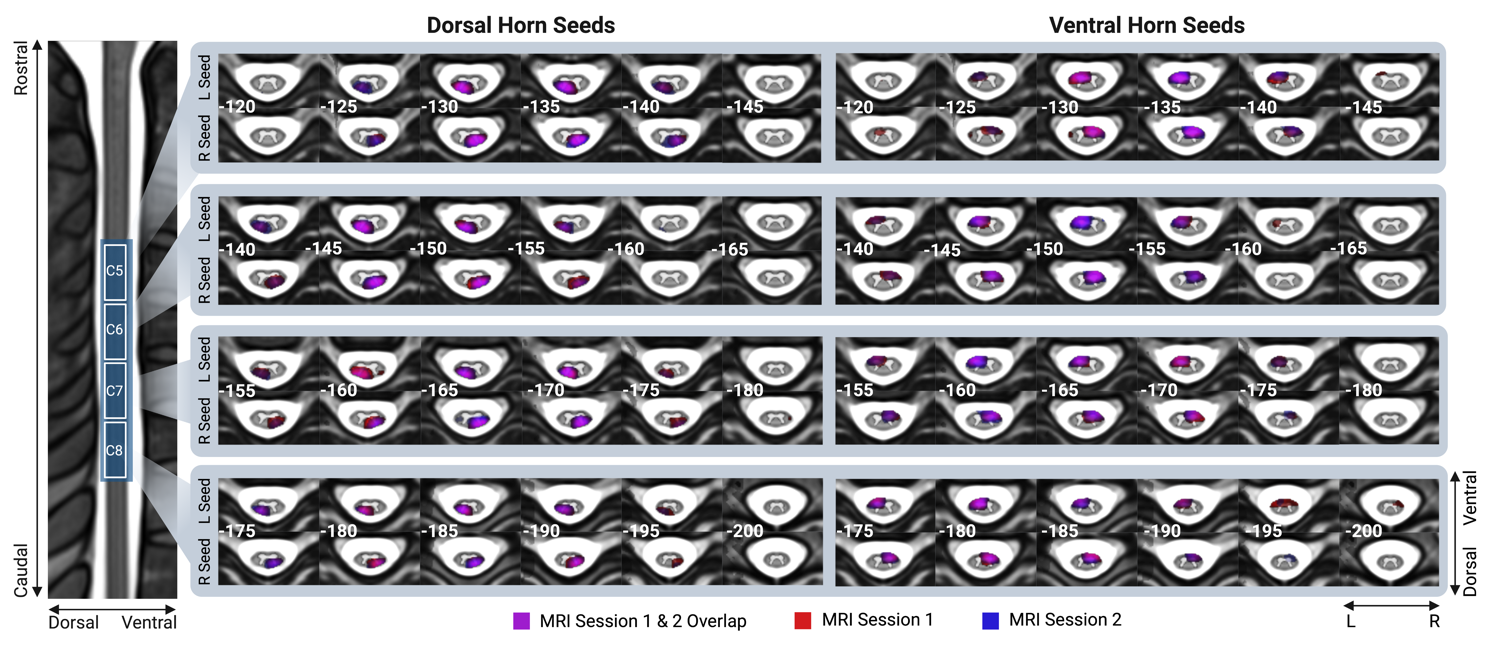


Supplementary Figure 4. Resting-state networks obtained from voxelwise connectivity analysis for each of the four horns (ventral/dorsal and left/right) of segmental levels C5-C8. Networks observed on MRI session 1 (red) and 2 (blue), as well as their overlap (purple) are displayed. Axial slices are marked with the *z* MNI coordinate. Each resting-state map was thresholded at *p*< 0.003 (*p*= 0.05, Bonferroni corrected for 16 individual seed regions).


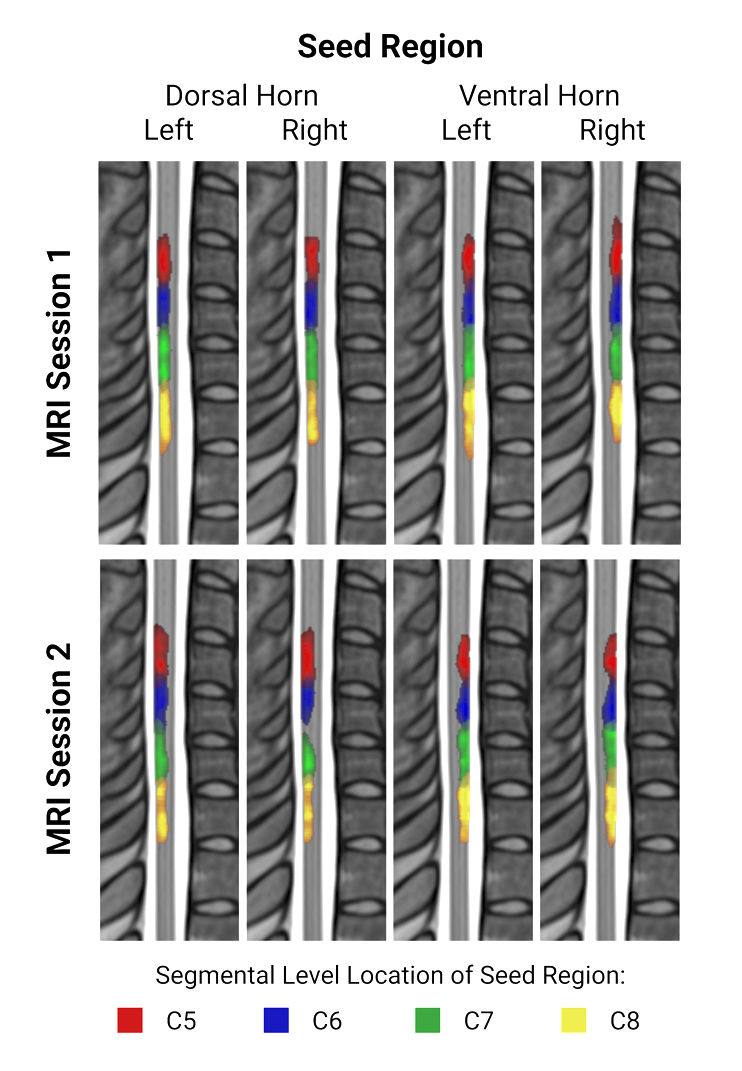


Supplementary Figure 5. A sagittal view (x = 0) of resting-state networks obtained from voxelwise connectivity analysis displaying sessions 1 (top row) and 2 (bottom row). Colours represent the location of seed regions used to estimate resting state networks (red = C5, blue = C6, green = C7, yellow = C8). Each resting-state map was thresholded at *p*< 0.003 (*p*= 0.05, Bonferroni corrected for 16 individual seed regions).


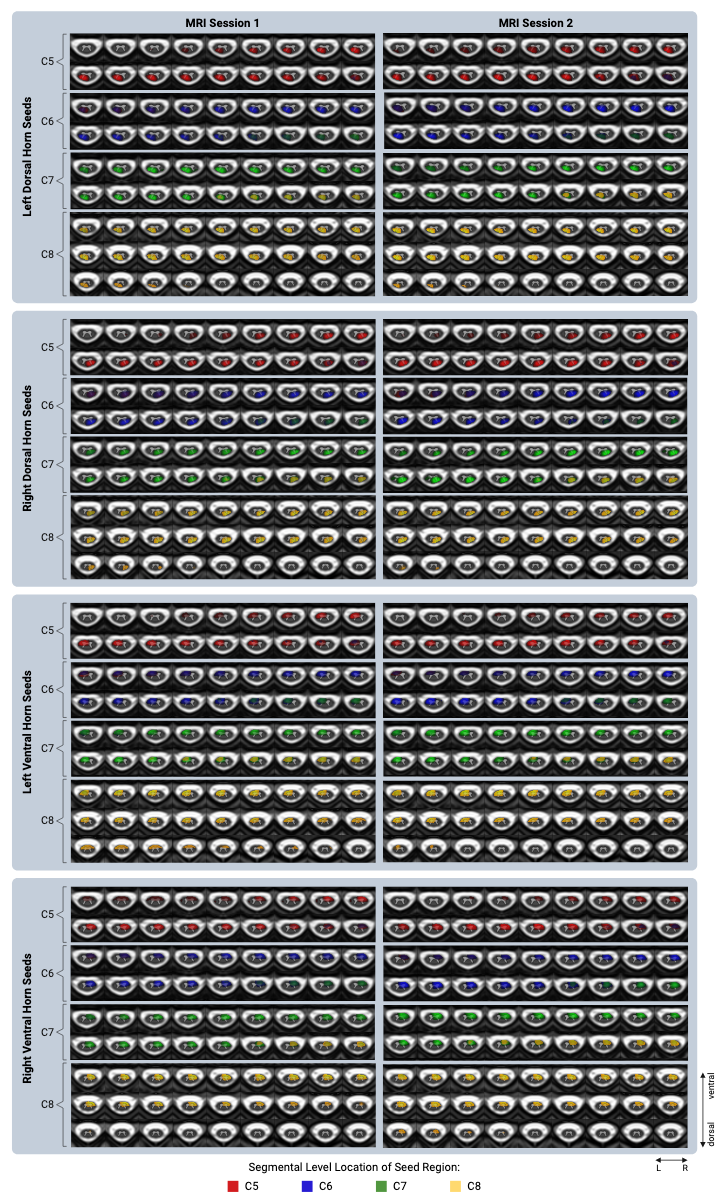


Supplementary Figure 6. A detailed breakdown of resting-state networks obtained from voxelwise connectivity analysis for each of the four horns (dorsal/ventral and left/right) of segmental levels C5-C8. Networks observed on MRI session 1 (left panel) and 2 (right panel) are displayed. Colours represent the location of seed regions used to estimate resting state networks (red = C5, blue = C6, green = C7, yellow = C8). Each resting-state map was thresholded at *p*< 0.003 (*p*= 0.05, Bonferroni corrected for 16 individual seed regions).

# Seed-to-seed connectivity analysis for data acquired during MRI session 2

## Methods

Seed-to-seed connectivity analysis of data acquired during MRI session 2 was performed identically to that of MRI session 1 (described in the main text). Briefly, Pearson correlations were computed between each pair of seed regions at subject-level. The resultant correlation coefficients were *Z*-transformed. Statistical significance at group-level was assessed using a one-sample *t*-test. A positive false discovery rate (FDR) was used to account for multiple comparisons (thresholded at *p* < 0.05).

## Results

A correlation matrix depicting cervical spinal cord connections is shown in Supplementary Figure 4. Overall, the pattern of results was similar to that observed on session 1. On average, within segment, the strongest statistically significant positive correlations were observed within hemicord (i.e. ipsilateral DH-VH), followed by VH-VH and DH-DH connections, and DH-VH connections between hemicords (i.e. left DH – right VH, right DH – left VH). Weaker but statistically significant positive correlations were also observed between neighbouring segments, including DH-DH, VH-VH, as well as within and between hemicords. Finally, negative correlations were observed between the right VH of segment C8 and both left and right DH of segments C5 and C6.


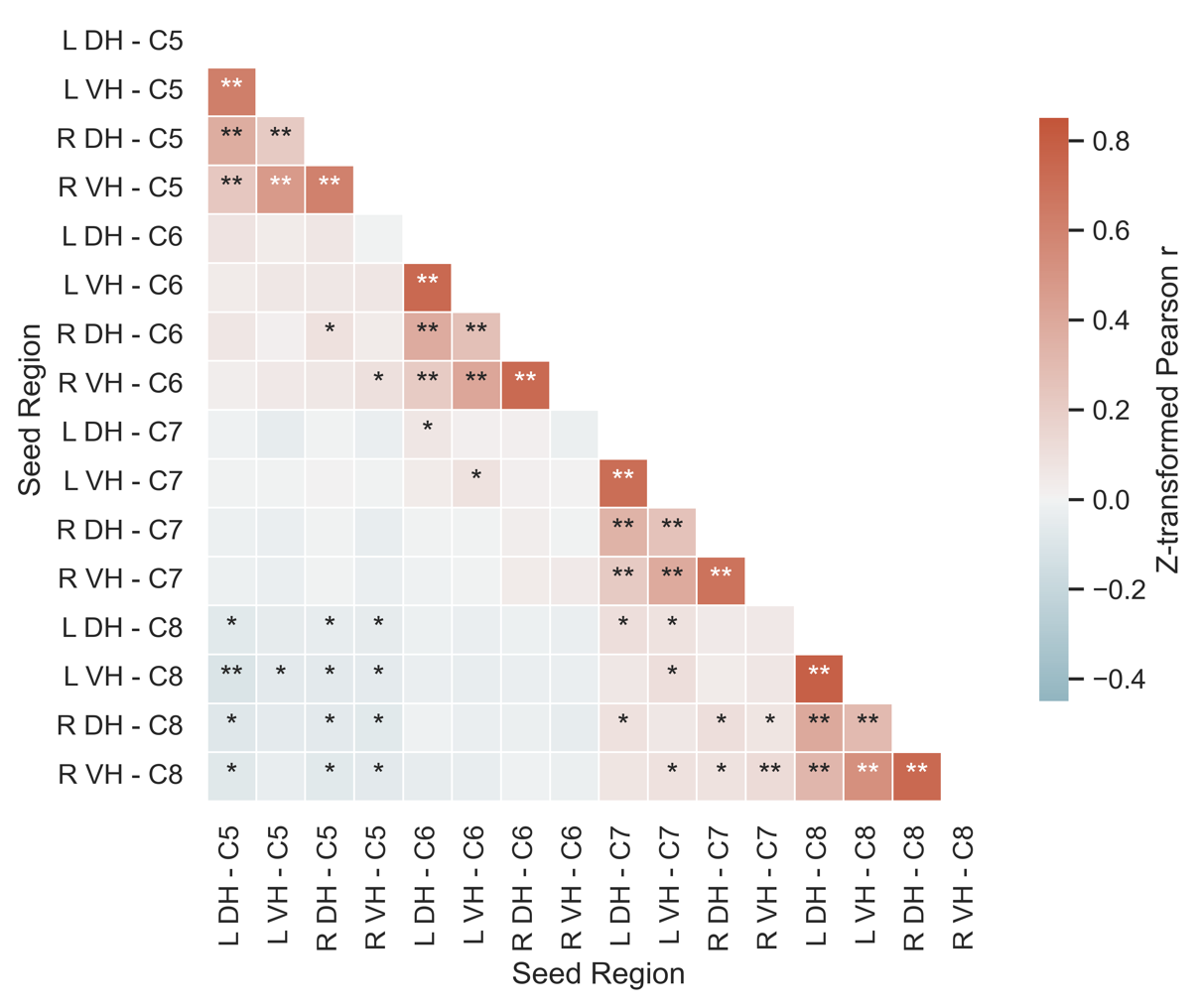


Supplementary Figure 7. Seed-to-seed correlation matrix obtained from session 2 data displaying z-transformed Pearson *r*.

DH = Dorsal Horn, L = Left, VH = Ventral Horn, R = Right.

*p < 0.05, **p < 0.001

# Seed-to-seed connectivity analysis on unsmoothed data

## Methods

Seed-to-seed connectivity analysis was repeated on unsmoothed data to unsure that spatial smoothing did not account for the observed results. All preprocessing steps were repeated identically as outlined in the main text with the exception of omitting spatial smoothing. Pearson correlations were computed between each pair of seed regions at subject-level for each session. The resultant correlation coefficients were *Z*-transformed. Statistical significance at group-level was assessed using a one-sample *t*-test. A positive false discovery rate (FDR) was used to account for multiple comparisons (thresholded at *p* < 0.05).

## Results

Correlation matrices depicting connections observed during sessions 1 and 2 are shown in Supplementary Figure 3 and Supplementary Figure 6, respectively. Overall, the patterns of results were similar to those observed on spatially smoothed data, confirming that the observed findings were not an artefact of spatial smoothing.


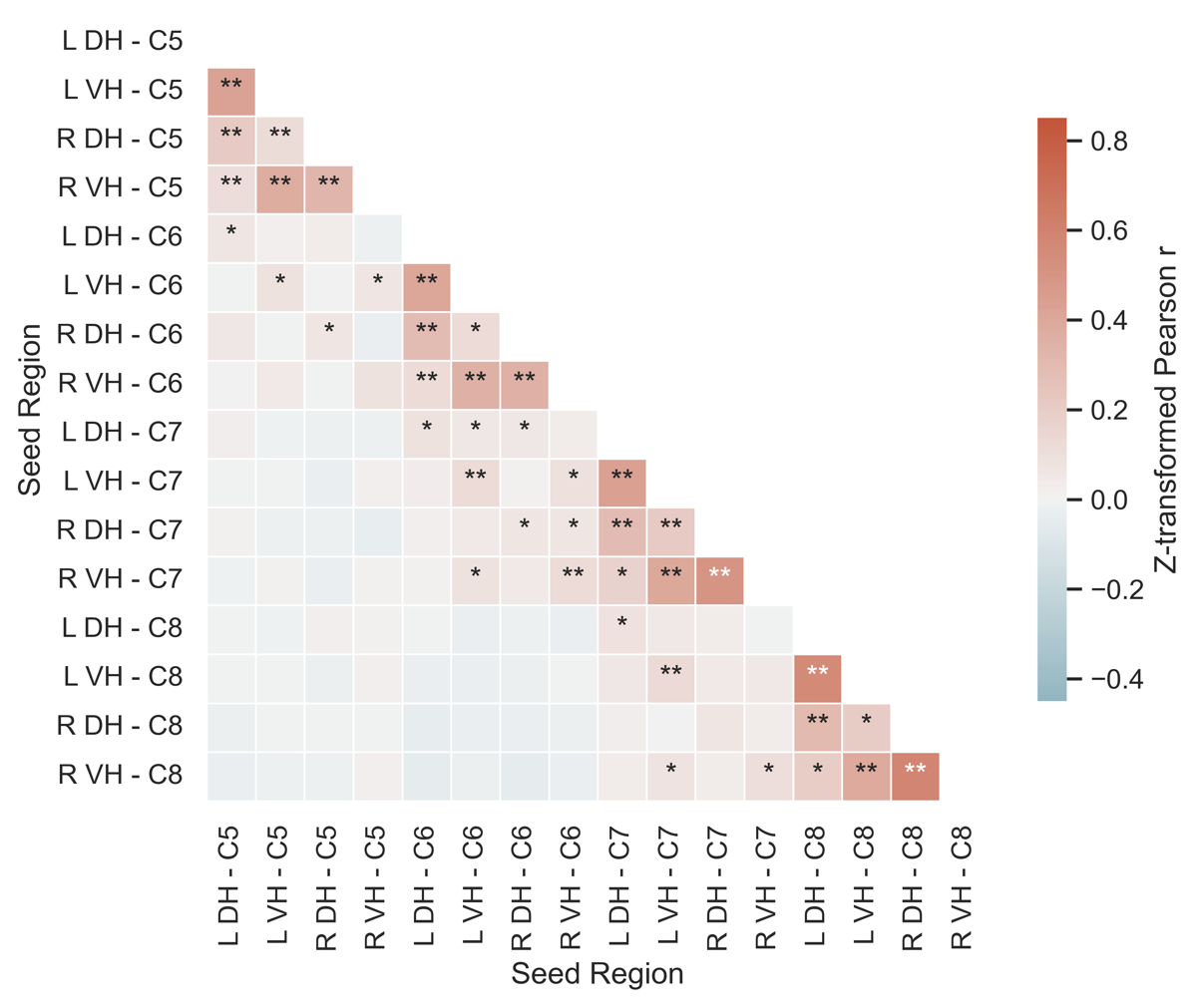


Supplementary Figure 8. Seed-to-seed correlation matrix obtained from unsmoothed data from session 1 displaying z-transformed Pearson *r*.

DH = Dorsal Horn, L = Left, VH = Ventral Horn, R = Right.

*p < 0.05, **p < 0.001


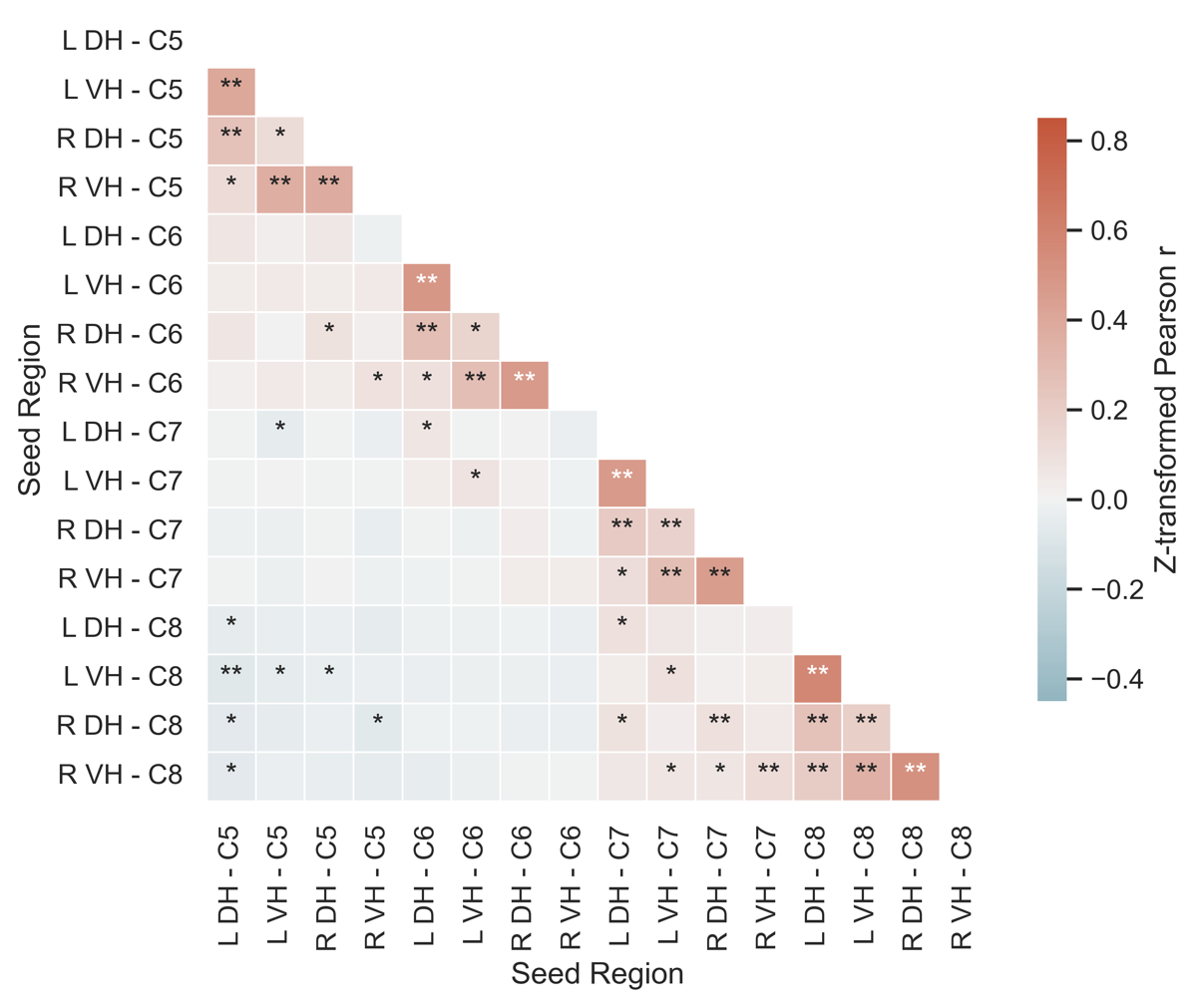


Supplementary Figure 9. Seed-to-seed correlation matrix obtained from unsmoothed data from session 2 displaying z-transformed Pearson *r*.

DH = Dorsal Horn, L = Left, VH = Ventral Horn, R = Right.

*p < 0.05, **p < 0.001

# Association between connectivity and ICC

## Methods

To explore whether connectivity strength and reliability by correlating ICCs obtained by comparing z-scored from seed-to-seed analysis across sessions with z-scored from seed-to-seed analysis averaged over the two sessions.

## Results

We observed a positive correlation between those variables (r = 0.43, p < 0.001). In line with similar observations made recently in brain fMRI (Tozzi et al., 2020), edges showing low reliability tend to be weak but edges characterised by good reliability are not necessarily strong (see Supplementary Figure 8).

*
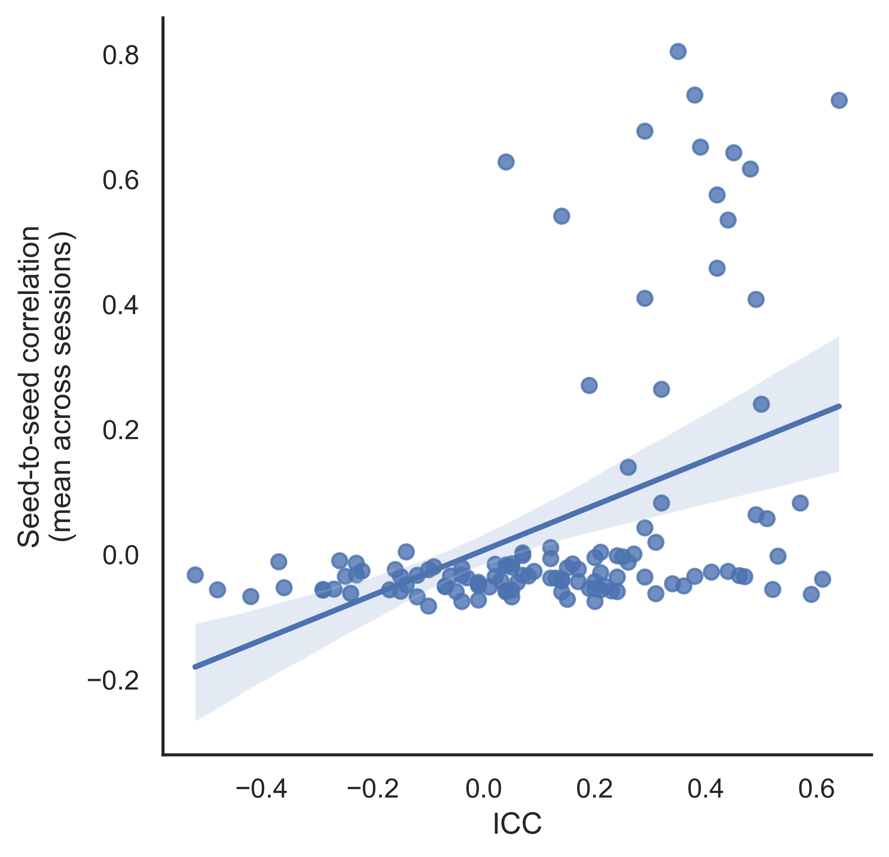
*

Supplementary Figure 10 Correlation between ICC (x-axis) and *z*-score of correlation strength obtained from seed-to-seed analysis averaged between sessions (y-axis).

# References

Tozzi, L., Fleming, S. L., Taylor, Z. D., Raterink, C. D., & Williams, L. M. (2020). Test-retest reliability of the human functional connectome over consecutive days: Identifying highly reliable portions and assessing the impact of methodological choices. *Network Neuroscience*, *4*(3), 925–945. https://doi.org/10.1162/netn_a_00148

Winkler, A. M., Ridgway, G. R., Webster, M. A., Smith, S. M., & Nichols, T. E. (2014). Permutation inference for the general linear model. *NeuroImage*, *92*, 381–397. https://doi.org/10.1016/j.neuroimage.2014.01.060
